# Development of polyester filters with polymer nanocomposite active layer for effective dye filtration

**DOI:** 10.1038/s41598-022-04829-4

**Published:** 2022-01-19

**Authors:** Mariia Pasichnyk, Jana Gaálová, Peter Minarik, Miroslava Václavíková, Inna Melnyk

**Affiliations:** 1 V.O. Sukhomlynskyi National University of Mykolaiv, 24, Nikolska, Mykolaiv, 54030 Ukraine; 2 Institute of Chemical Process Fundamentals of the CAS, v.v.i., 135, Rozvojova, Prague, 16500 Czech Republic; 3 Charles University, 5, Ke Karlovu, Prague, 12116 Czech Republic; 4 Institute of Geotechnics Slovak Academy of Sciences, 45, Watsonova, Kosice, 04001 Slovak Republic

**Keywords:** Composites, Materials chemistry, Pollution remediation

## Abstract

Organic dyes such as methyl orange (MO) and methylene blue (MB) are widely used in different industries and have become one of the leading emerging water contaminants. The purpose of the current research is to develop new polymer nanocomposite filters for the effective elimination of the dyes, which are non-biodegradable and not efficiently removed by traditional treatment methods. New padded and covered filters were produced applying polystyrene-acrylic/ZnO nanocomposite on the polyester surface by blade coating and one-bath pad methods. Principal results determined by SEM analysis confirm that functionalised layer can create unprecedented function of filter textile material depending on the way of treatment. Due to the modification, the surface area increased from 5.9 for untreated polyester to 85.2 (padded), 44.6 (covered) m^2^/g. The measured pore size of produced filters is around 3.4 nm, which corresponds to the mesoporous structure. Our study reported effective filters with the rate of MB and MO removal efficiencies up to 60%. Moreover, a colourless reduced form of MB—leuco-methylene blue (LMB) could be created. The functionalised layer of the developed filters through hydrogen bonding between the –OH groups of styrene-acrylic molecules and the –N(CH_3_)_2_ groups on LMB can stabilise LMB.

## Introduction

Water pollution by industrial effluent like organic dyes is now one of the critical issues worldwide. The growing concentration of dye pollutants in water is hazardous. It causes significant environmental problems by reducing the photosynthetic activity and oxygen enrichment of aquatic organisms by decreasing light transmittance^1^.

Various physicochemical approaches have been made to remove organic pollutants from water, such as chemical precipitation^2^, filtration^3^, coagulation and flocculation processes^4^, use of oxidizing agents^5^ and photocatalytic technology^6^. Filtration is one of the easiest ways to remove pollutants from wastewater. The development and implementation of new types of filters with higher filtering capabilities have great importance^4^.

Polymer nanocoated textiles have shown good results as water filters due to the material’s high physical and mechanical resistance^7^. By controlling the finishing processes of the textile material, it is easy to create the required porosity, increase filter life, and save material properties. Nanofillers in the polymer composition can provide high durability for fabrics because nanoparticles have a large surface area-to-volume ratio and high surface energy^8^, thus presenting better fabric affinity and increasing durability^9^. Furthermore, the photoactivity of modified textiles was investigated in the decontamination of organic dyes^10,11^ and degradation of gaseous pollutants^12^. Moreover, the nanoparticles can penetrate certain parts of the substrate, such as pores, holes, and crevices, and mechanically lock to the substrate. The voids between the nanoparticles can be utilised as filtration channels to increase filtration performance.

Currently, the production of photoactive textiles incorporated with photocatalytic particles is of great interest—considerable research has been devoted to preparing and investigating dip-coated textile﻿s^13^. Han and Bai immobilised different layers of titanium dioxide onto the surface of polypropylene fabric. They confirmed that MO dye solution degradation under UV and visible lights could be greatly improved with one layer of titanium dioxide coating. Such fabrics can also be used for the photocatalytic oxidation of phenol from water^14^.

Zhang and Zhu immobilised Fe-doped TiO_2_ on the surface of polyamide fabric under hydrothermal conditions. The coating improved photocatalytic activity against MB^15^. Ag-TiO_2_ was synthesised by photo-reducing Ag^+^ ions to Ag metal and then coated on cotton fabric using the pad-dry-cure method. The coated fabric showed high efficiency against MB under normal laboratory environment conditions^16^.

In the present work, a polymer composition for the finishing of polyester textile was created by mixing acrylic copolymer, a cross-linked melamine agent, and ZnO nanoparticles. The polyester material was treated by polymer composition using the coating and padded methods. The developed textile material was used for the filtration of organic dyes from the model wastewater.

## Results

The structure and properties of the yarn and fabric were optimised considering the characteristics of filter fabrics (Table 1). Different surface treatments of the polyester led to different types of etching due to chemical modification. The surface becomes smooth, which leads to deterioration of wettability.

**Table 1:** Structure characteristics of filters.

	Mass of filter, g	Mass of composition, g	Thickness, mm	Area density, g/m^2^	Bulk density, g/cm^3^
Polyester	0.72	–	0.315	128	2.28
Padded polyester	0.87	0.14	0.333	155	2.61
Covered polyester	1.08	0.36	0.340	192	3.17

**Figure 1:**
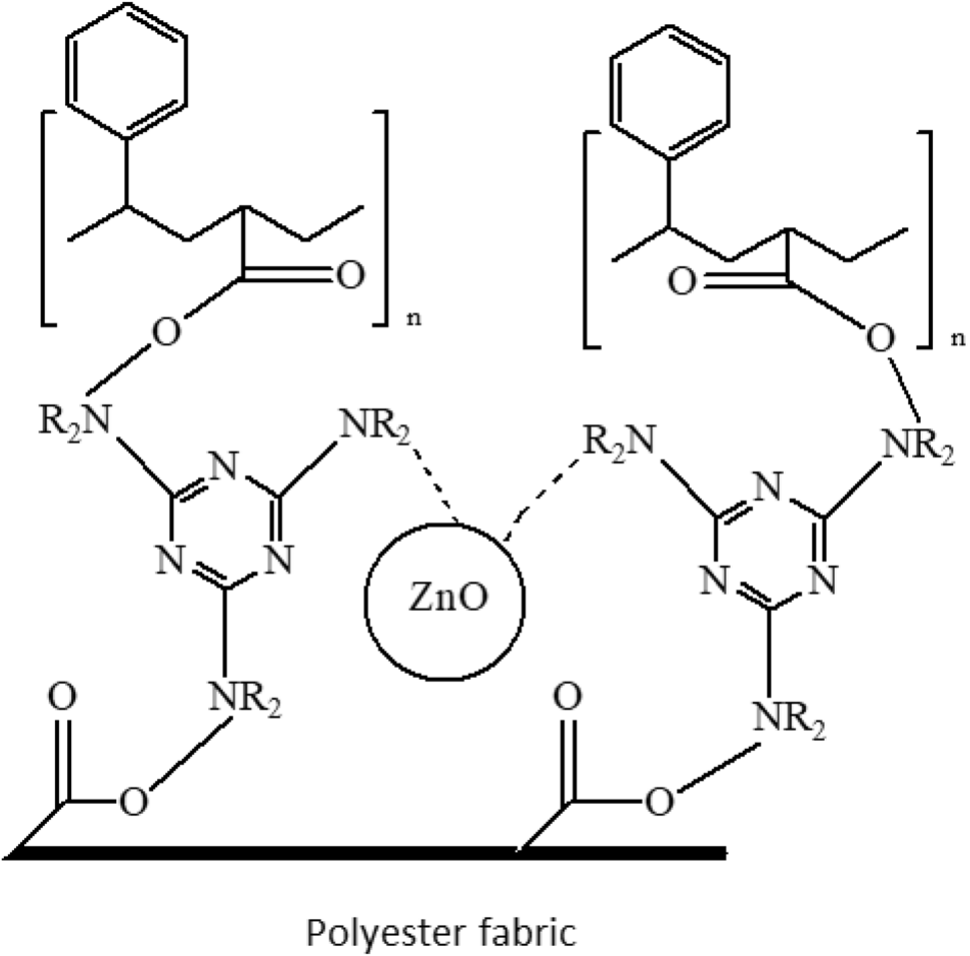
Scheme of the polyester finishing by polymer nanocomposition.

The finishing process happens due to the mechanism shown in Fig. 1. Partially esterified melamine is linked covalently to polyester fabrics and a styrene-acrylic binder by curing at 140–150 °C through trans-amidation. At these temperatures, the primary amino groups of the melamine react with the carboxylic acids groups of styrene-acrylate and the accessible ester groups at the surface of the polyester fibre.

The polymer matrix and textile material can stabilise nanoparticles. Such stabilisation provides fixing the nanoparticles to each other or fixing the particle layer to the support layer. This treatment provided a diversity of textile materials and pore geometries. High flow resistance is possible during filtration if the particles used as fillers are selected by size.

**Figure 2:**
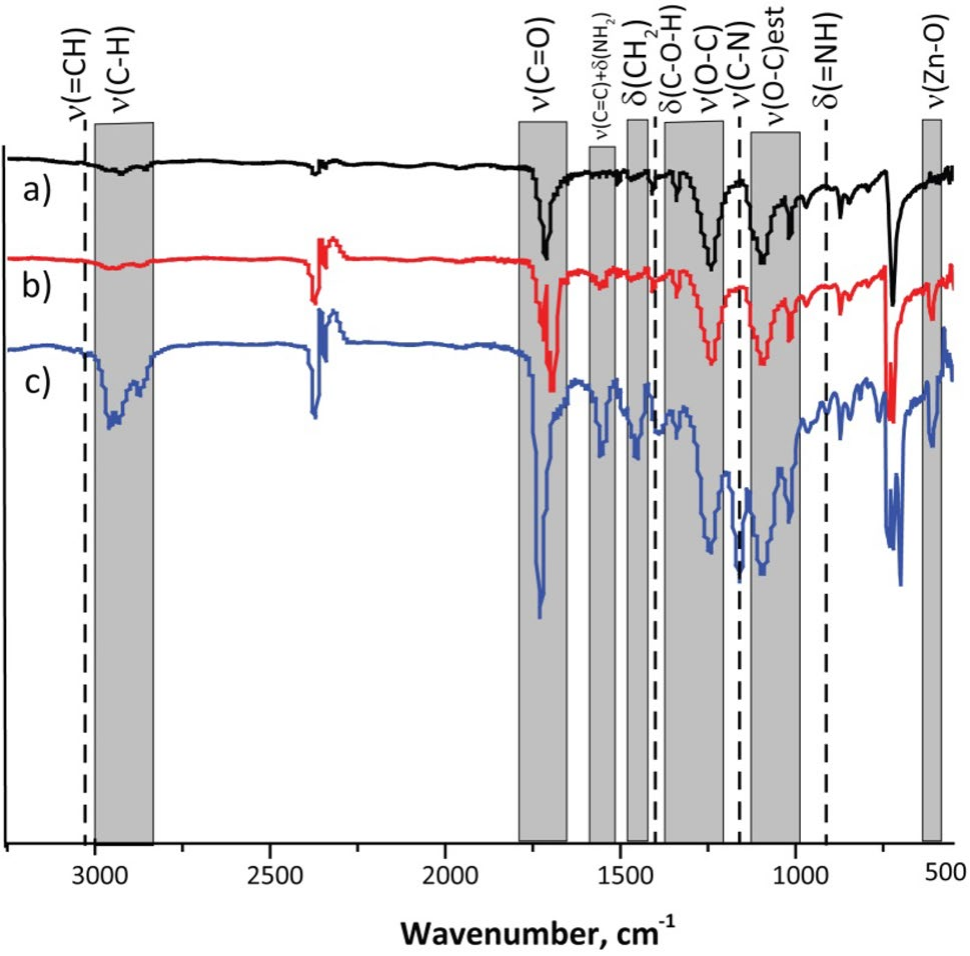
Fourier transform infrared spectra (FTIR) of (**a**) polyester, (**b**) padded polyester, (**c**) covered polyester.

The surface chemical modifications of finished polyester fabrics were determined by an FTIR analysis in the range of 500–4000 cm^−1^. Figure 2 shows the FTIR spectra obtained for the untreated polyester, padded polyester and covered polyester. In untreated polyester, the presence of ester, alcohol, anhydride, and aromatic rings is seen. Absorption bands at 2924 and 2848 cm^−1^ are characteristic for ν_s,as_(C–H) bonds from alkyl chains, band at the wavelength of 1710 cm^−1^ is related to the stretching vibration of the carbonyl group ν(C=O) from acid, low-intensive band at 1460 cm^−1^ is corresponded to δ(CH_2_) vibrations, two bands at 1338 and 1239 cm^−1^—to ν(O–C) from acid as well, and two peaks at 1093 and 1012 cm^−1^ to ν(O–C) from ester, the peak at 700 cm^−1^ is attributed to the bending and ring puckering vibrations of the C–H on the benzene rings^17^. This is a reason that alcohol and anhydride remained as residual reactants in the polyester. The carboxyl, ester, anhydride, and alcohol groups showed that the polyester fabric was not pure. The intensity and position of absorption bands for the padded polyester were not changed much. A shift of absorption band of ν(C=O) vibration is seen at 1692 cm^−1^, indicating the adhesion of acrylic acid to the surface and the introduction of new carboxyl groups^18^. This type of composite polyester showed absorption peaks at 2959 and 2870 cm^−1^, due to the stretching vibration of C–H groups and a low-intense band at 1558 cm^−1^, due to the bending vibrations of –NH_2_ groups and stretching ν(C=C) from the rings. IR spectrum of the covered polyester contains a band at 3030 cm^−1^ attributable to ν(=CH), the wavelength of C=O shifts to the left (1729 cm^−1^), indicating an increase in the absorption intensity due to the introduction of acidic groups. Also, the proportion of the O-and N-containing groups such as C=O, –C–OH, –COOH, and –NH_2_ increased on the surface of the treated fabric, and, as result, the intensity of absorption bands at 1728, 1558, 1449, 1391 cm^−1^. There are also two new absorption bands at 1154 cm^−1^ and 908 cm^−1^, which correspond to stretching ν(C–N) and bending wagging vibrations of –NH_2_ and =NH, respectively. These results could be a result of the active groups of the polymer nanocomposite reacting with the active –O–C=O (carboxylic) groups of the polyester fabric, resulting in the formation of oxygen-containing polar groups on the fabric surface. The introduction of oxygen-containing polar groups on the fabric surface changes the nature of the surface. The intensity of the peaks for covered polyester was sharpened between the untreated and padded, indicating that acrylic acid bonded with polyester fibres in the process. The observed absorption bands (Fig. 2b,c) at 605 cm^−1^ result from the stretching vibrations of Zn–O in the covered and padded polyester samples, indicating that ZnO nanoparticles are present^19^.

### The structure of the surfaces

**Figure 3:**
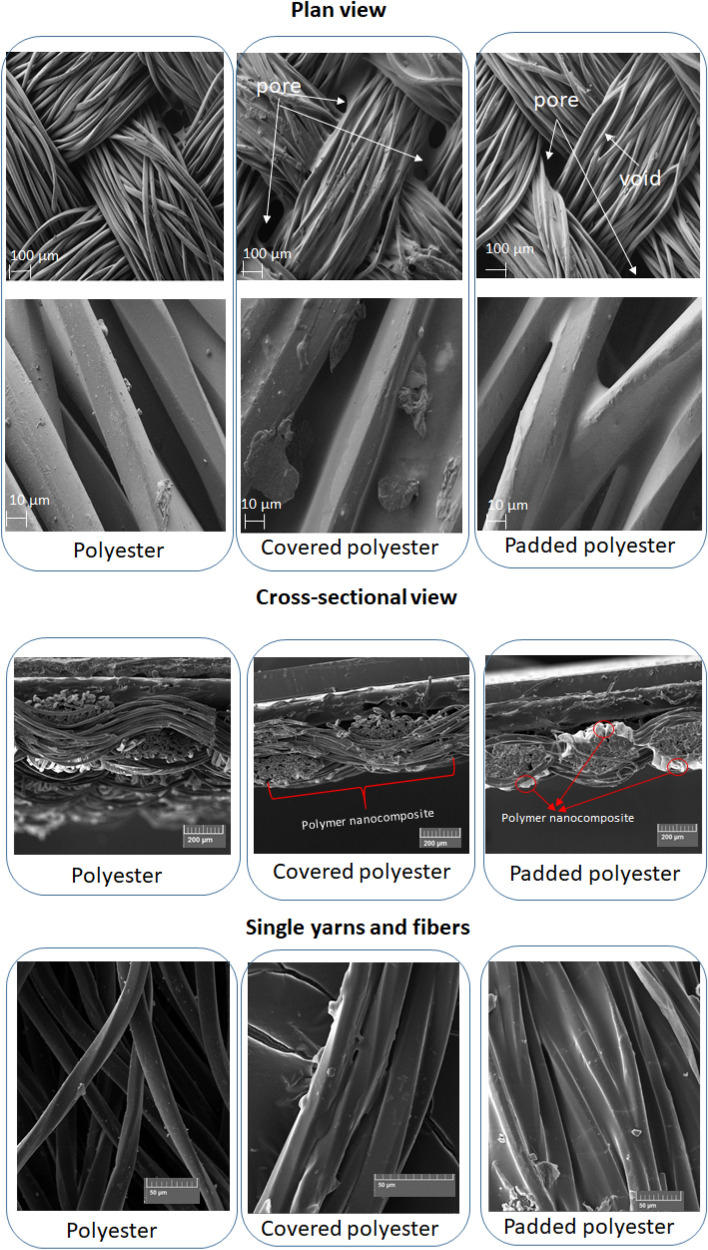
SEM images of the samples.

The structure of the coated surfaces was characterized by scanning electron microscopy (SEM). The SEM images were used to investigate the change in the surface morphology of the untreated, padded and covered polyester fabric, as shown in Fig. 3. The surface of untreated polyester is smooth and distinct. The yarns were layered naturally with porous structures. The polyester surface becomes rough due to the applying of the polymer nanocomposite. The surface of the covered polyester consists of big particles of unmixed acrylic dispersion, that's why pores are not regular. The padded polyester shows a drastic change in the fibre surface morphology, with the presence of voids and pores. Particularly, between the neighbouring yarns, empty spacing’s bridged by the attached polymer nanocomposite conductive networks.

The untreated polyester yarns are quite fluffy, clearly can be seen the single fibres in yarn. By applying polymer nanocomposition, single fibres are glued, which decreases the yarn diameter. Moreover, it can be noticed, polymer nanocomposite between adjacent yarns.

The plane view SEM images show that the structure of treated polyester is relatively bulky, and the structure of the polymer composition is relatively dense. Due to the fact that polymer composition consists of ZnO nanoparticles, on the surface of textile can be formed cross-linked structure which has cross-linked units with components of composition and polyester fibres^20^. As a result, the structure became crisscross and intertwined, so the filter pore size decreased. This factor can’t be ignored, as the combination of polymer nanocomposite surface and polyester substrate will increase the filtration resistance and improve the filtration efficiency. Produced layer decreases the possibility for the penetration of the dye molecules into the fibres and lets them rather adsorbed on the surface.

According to the cross-section SEM images, a smooth film coating uniformly dispersed on the surface and around the fibres on the top of the fabric in covered polyester. Polymer nanocomposite adhered to the textile but did not penetrate deeply into the textile. In padded polyester, polymer nanocomposition was formed both on the yarn surface and inside the adjacent yarns. The surface consists of the active layer, but the opposite side of the polyester also includes parts of polymer composition with a maximum thickness of ~ 50 μm.

**Figure 4:**
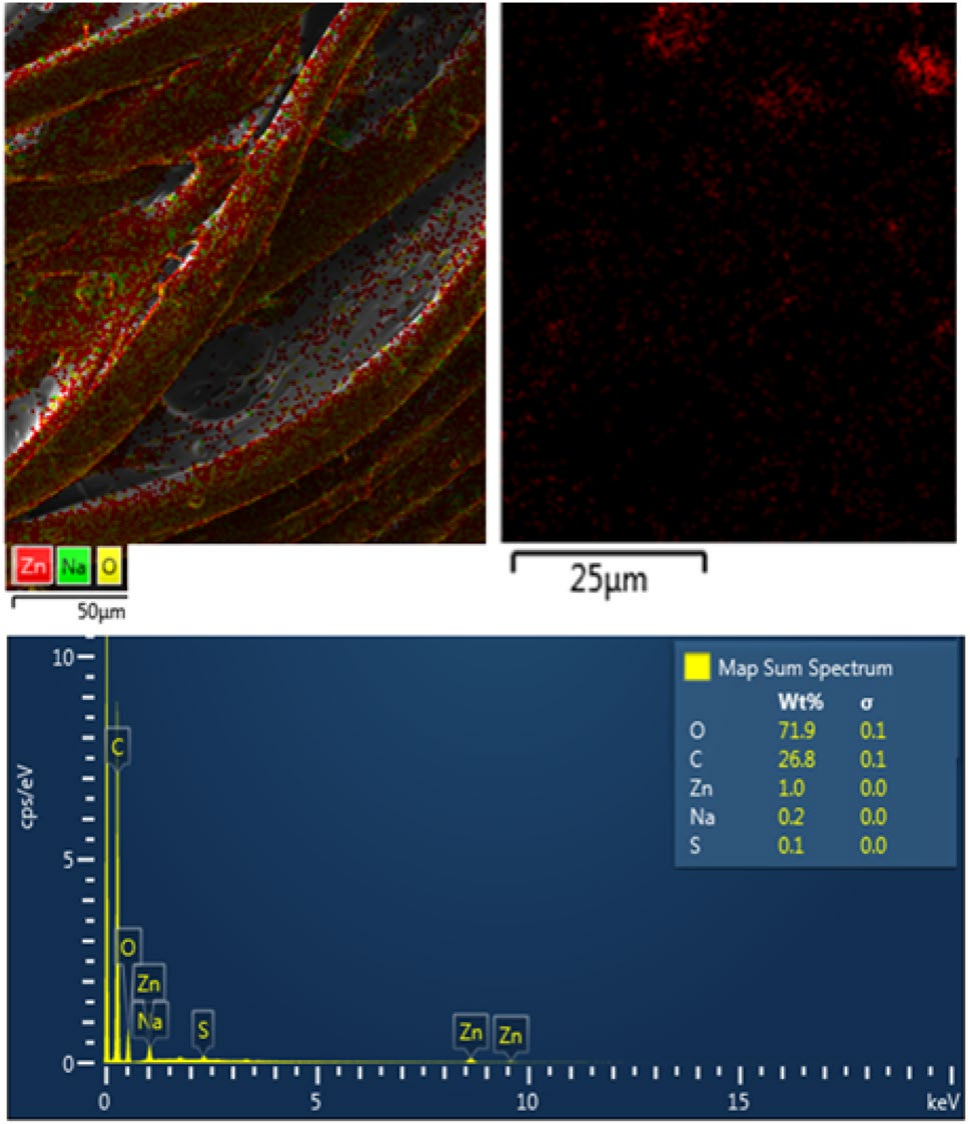
EDS layer images of the padded polyester.

The result of the elemental mapping performed on the surface of the padded polyester fabric sample is shown in Fig. 4. The nanoscale ZnO particles were well distributed on the surface of polyester. The particle size plays a primary role in determining their adhesion to the fibre. It is reasonable to expect that the largest particle agglomerates will be easily removed from the fibre surface. In contrast, the smaller particles in the polymer composition will penetrate deeper and adhere strongly to the fabric matrix.

### Hydrophobicity

**Figure 5:**
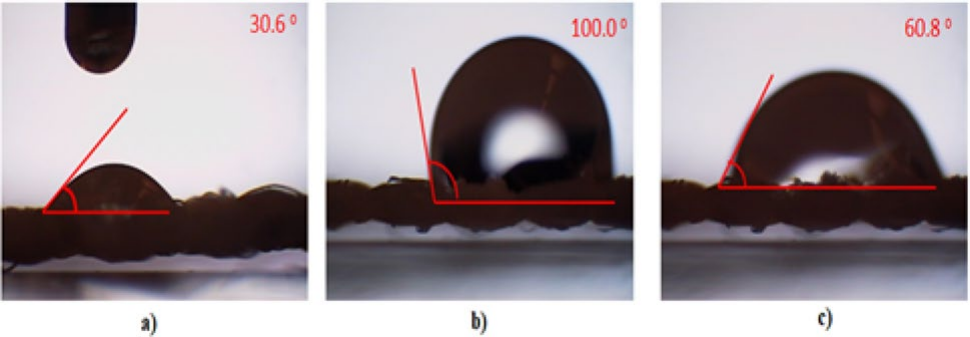
Optical image of water contact angle (**a**) untreated polyester, (**b**) covered polyester, (**c**) padded polyester.

The hydrophobicity was improved by modification of the filters using the polymer composition. The results of the studied filters are shown in Fig. 5. When the polyester textile material was treated with the polymer composition, the contact angle was dramatically increased, indicating that the hydrophobicity of the surface was greatly improved.

Untreated textile material exhibited poor hydrophobicity with a water contact angle (WCA) of 30.6°. Covered polyester had good hydrophobic properties, with a measured WCA of 100.0°. Padded polyester had poor hydrophobicity with a WCA of 60.8°.

These findings contradict previous studies of polymer nanocomposite films formed on glass surfaces. The polymer film created a smooth surface on the glass^21^. Polyester fabric is characterised by hairiness which forms an uneven surface. As the textile fibre is coated with the polymer nanocomposite film, the texture created on the surface of the textile characterise by hierarchical structure and increased surface roughness^22^, which significantly improves the hydrophobic properties of treated polyester fabric^23^. The primary reason treated filters become hydrophobic is that polluted water droplets are stably supported on the hierarchical structure of the filter surface, and dyes can form pockets in the interface.

### Water absorption test

**Figure 6:**
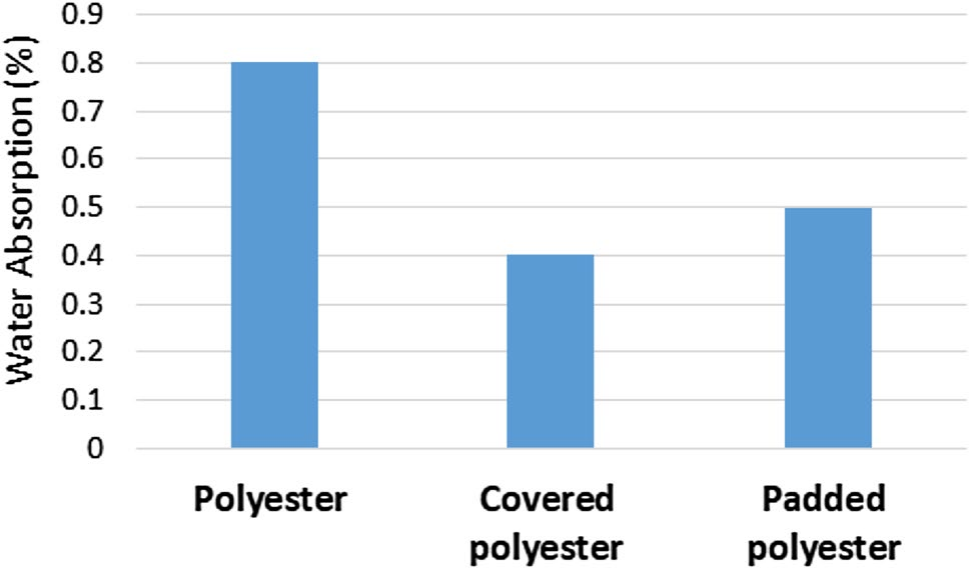
Water absorption test.

Water absorption (retention) test results for the filter materials are given in Fig. 6. The standard polyester fabric absorbs a minimal amount of water (0.8%). Water absorption decreases dramatically for padded polyester. Polymer nanocomposite applied to the polyester surface made a significant difference in the water absorbency of the filter media. The differences in water absorption values of padded and covered polyester occurred because of the polymer’s low adsorption property due to its ability to decrease pore diameter and change fabric structure.

### Low-temperature nitrogen adsorption–desorption isotherms

Figure S1 shows the nitrogen adsorption–desorption isotherms obtained at 77 K for polyester textile material prepared with different treatment techniques. The shape of isotherms is not standard, as developed material consists of polyester support and polymer nanocomposite layer. In this way, it is difficult to attribute the exhibit steep to any type, as more evident than in the material can happen combination of several types. However, it can be noticed that the development of mesoporosity is indicated by the pronounced hysteresis loops that appear for samples. Covered polyester shows a wide hysteresis loop, and the desorption curve is steeper than the adsorption branch, indicating that the samples have various pore types and pore diameter distributions. Padded polyester characterised with narrow hysteresis loop. Meanwhile, untreated polyester shows open-wedge pores.

The surface areas of the filter available to the nitrogen vapour were calculated according to the Brunauer, Emmett and Teller (BET) equation. The calculated surface area values are presented in Table 2. It is pointed out that the surface area available to the nitrogen vapour is greatly dependent upon the way of polyester treatment. Polymer nanocomposite film was also tested and showed a non-porous structure with a low specific surface value of 8.7 m^2^/g. In addition, the BET area of the polyester textile material increased significantly after applying polymer nanocomposite, indicating complete pore filling. The surface area increased from 5.9 for untreated polyester to 85.2 (Padded), 44.6 (Covered) m^2^/g. The surface area of treated polyester shows the presence of mesoporous (pores with characteristic dimensions ranging from 2 to 50 nm). It follows that padded polyester reveals the largest specific surface area. Furthermore, the aforementioned sample reveals also the highest value of the total pore volume, V_tot_. In addition, the reduction of the total pore volume and the micropores volume was observed in covered polyester. These results indicate that polymer nanocomposition block the pores, which caused a decrease of the above-mentioned parameters. Moreover, the difference in the surface area between padded and covered samples was attributed to the different polymer nanocomposite content and way of treatment of initial polyester. Contrary, all tested samples show a negligible portion of micropores (i.e. pores smaller than 2 nm).

**Table 2:** Characteristics of the produced filters surfaces.

	Surface area S_BET_, m^2^/g	Total pore volume V_tot_, cm^3^/g	Pore size, nm
Polyester	5.9	0.036	3.4
Covered polyester	44.6	0.028	3.1
Padded polyester	85.2	0.065	3.4
Polymer composition	8.7	–	–

### Pore size distribution

The proper selection of filter material is essential for achieving efficient filtration. Figure S2 shows the distribution of the pore size.

The average pore size of untreated, covered and padded polyester was ~ 3.4 nm. Total pore volume was 0.036 cm^3^/g, 0.028 cm^3^/g, and 0.065 cm^3^/g, respectively. The pore size of covered polyester was smaller than untreated and padded polyester. Moreover, the average pore size of untreated polyester was the same as padded. Pore distribution for padded polyester ranged from 4.2 to 12.4 nm, indicating the pore size was not uniform. The surface of the polyester substrate consisted of a large number of fibres interweaving and piling up with each other, contributing to the formation of some large pores between the fibres. On the other hand, the accumulation of fibres on the surface may have resulted in the partial blockage of the pores and, therefore, some small pores formed^24^.

### Filtration experiment

**Figure 7:**
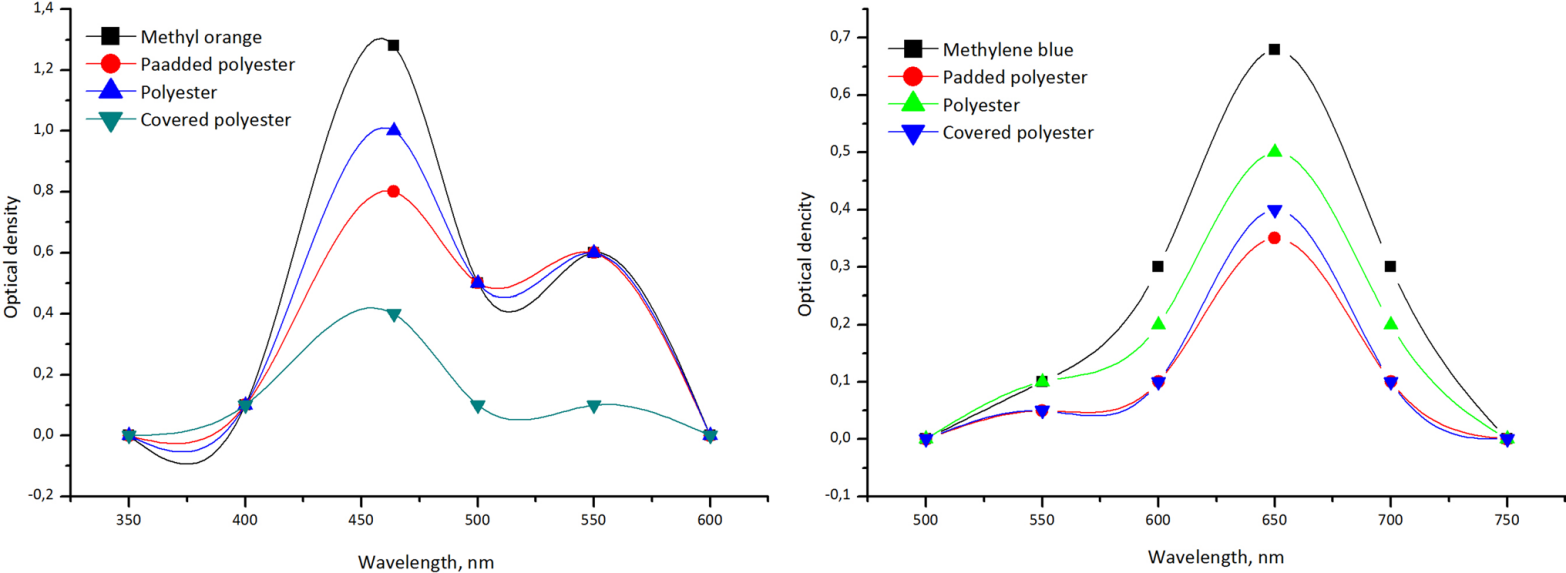
UV–Visible spectra of methylene blue and methyl orange before and after filtration.

The possibility of using these fabrics for filters was explored with MB and MO solutions. The size of the MB molecule is around 13.82 Å^25^. The MO molecules have a larger size ~ 26.14 Å^26^. Considering the length of the dye molecules and the dimension of the pores in the filters, organic dyes can easily enter into the pores. The concentration of MB decreased from 100 to 60 ppm using the padded polyester, and the concentration of MO declined from 100 to 40 ppm with the covered polyester (Fig. 7). These observations indicate that organic dyes of different nature can be effectively removed from the water using a suitably processed textile filter. It is evident that the covered technology almost totally encloses the pores. Meanwhile, the padded technology decreased the ratio between fibres and formed a solid covering of the pores.

### Filtration efficiency

It was observed that the movement of the dye particles typically deviated from the water flow, especially as they approached the fibre. During the filtration Brownian diffusion, the electrostatic and the gravity effects occurred. The electrostatic effect firmly attaches the particles to the surface of the fibres. Results are presented in Table 3.

**Table 3:** Filtration of organic dyes.

	pH ZPC	MB		MO	
		Equilibrium quantity that can adsorb on the filter (t = 120 s), mg/g	Removal rate (R), %	Equilibrium quantity adsorb on the filter (t = 120 s), mg/g	Removal rate (R), %
Polyester	6.81	0.69	20	0.52	15
Padded polyester	6.29	1.14	40	1.0	35
Covered polyester	6.65	0.81	35	0.92	60

## Discussion

**Figure 8:**
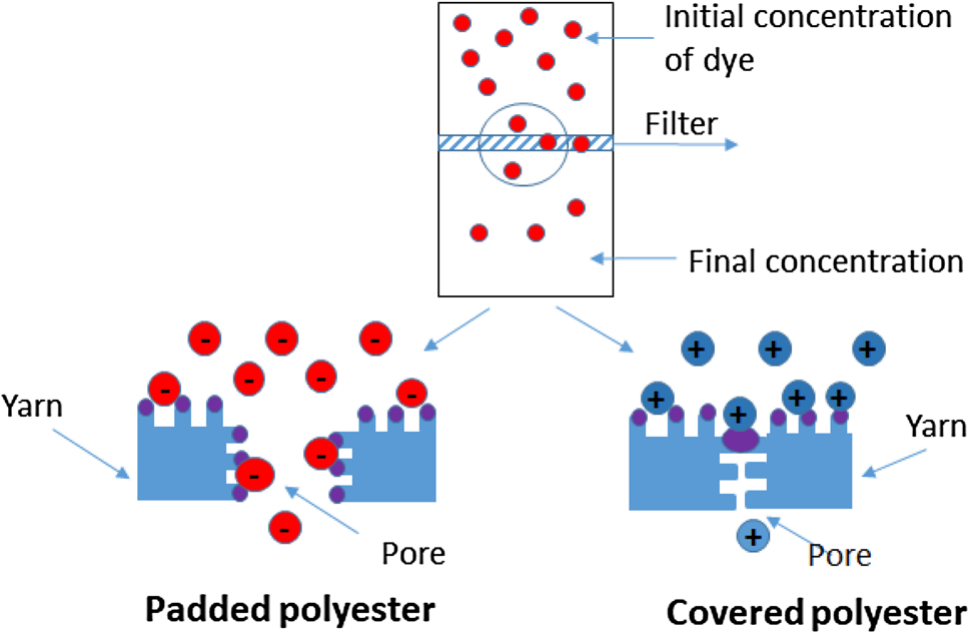
Scheme of produced filters.

Using technology to treat polyester fabric provides a unique method to lower the energy barrier between the polymer nanocomposition and the filter surface and thus increase the deposition of dye particles on the surface of the filter. Unionised dye molecules diffuse through the covered and padded filter because the polymer matrix and dyes are hydrophobic. Moreover, the pores are covered with a polymer composition in padded polyester, increasing the filter adsorption capacity. In covered polyester, all interactions happen on the surface of the filter. Furthermore, in covering technology, polymer nanocomposition covers the pores, which 'reduces its size by twofold (Fig. 8).

The pH of the dye solution was 6.00, which is below the pH of zero point of charge (pH ZPC) of the treated polyester surface. The surface of produced filters exhibited basic properties (Table 3). Decreasing the pH of the surface led to an increase in the concentration of H^+^ ions in the solution. Padded polyester with pH ZPC 6.3 releasing H^+^ ions induced a positive charge on the terminal nitrogen of MO, which helped adsorption through the anion-exchange mechanism. The maximum dye removal efficiency of MO using covered polyester could be attributed to the electrostatic attraction between positively charged surfaces (ZnOH^+^)^27,28^.

The removal efficiency of MB is lower than MO due to the bigger size of MB anions. Some MB anions could be excluded because of the filters’ sieve effect’. Others could be adsorbed on the surface of the filter by an electrostatic attraction. An ion-exchange mechanism was created between the nitrogen of the amino groups of MB, the nitrogen of the –NH_2_ group of melamine in the polymer composition, and the oxygen of the carbonyl group of the styrene-acrylate.

**Figure 9:**
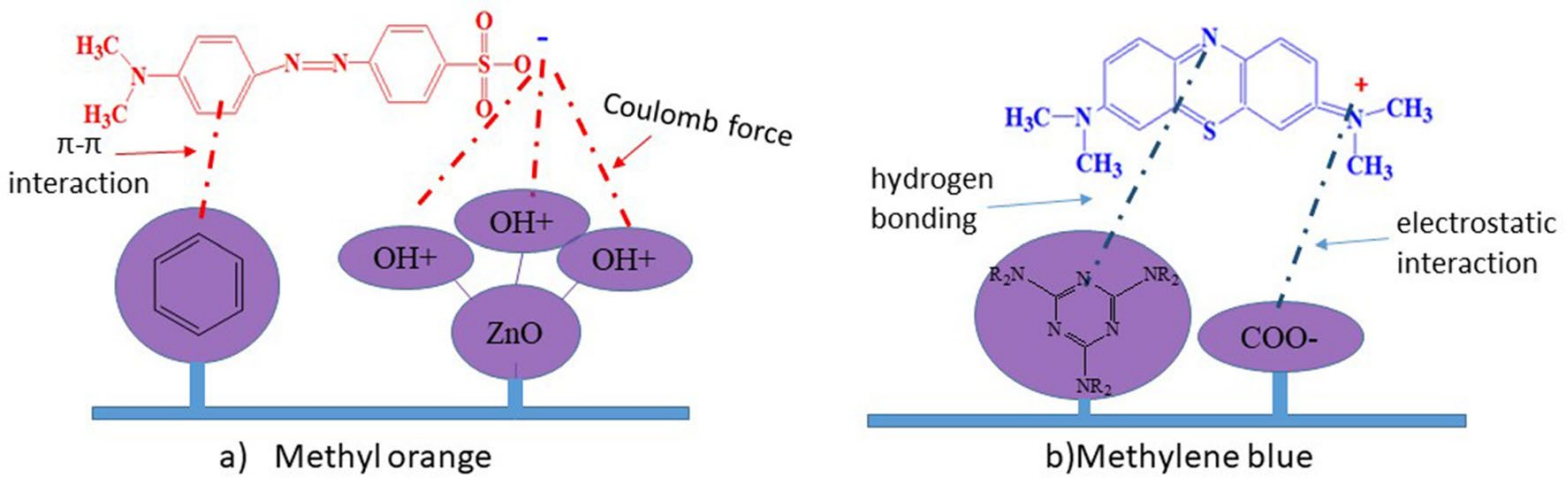
Possible mechanisms of interaction.

Figure 8 shows that the cationic nature of the dye ensures an attraction with the polymer covering. Based on the experimental results obtained, the mechanism of dye transport across the polyester filter can be interpreted as follows (Fig. 9):

Most researchers were focused on detecting the highly coloured form of MB; meanwhile, its colourless reduced form, leuco-methylene blue (LMB), has not been the subject of much interest. In this article, we report the formation of LMB, which was previously not considered when determining the adsorption properties of materials. Such observations are not surprising, as LMB is colourless and weakly absorbs in the near UV range and absorbs more strongly in the far UV (λ max = 256 nm).

Several works have reported the chemical transformation between the highly coloured oxidised form of MB and its stable colourless reduced LMB. The dye's colour changed from blue to colourless, corresponding to the hydrogenation of MB to LMB. MB to LMB was reduced using ascorbic acid^29,30^, acrylate media^31^, an ionic liquid^32^, and a nitrogen environment. MB is characterised by two main peaks. One is at 662 nm due to substituting the –N(CH_3_)_2_ group on the heteroaromatic ring (responsible for colour). The other at 292 nm is associated with localised bands of the unsaturated heteroaromatic system.

The filtration results with the developed filters show that the intensity of these peaks markedly decreased. Also, the spectra showed an increase in the peak at 246 nm, which is responsible for LMB formation. Filtration with covered polyester resulted in the disappearance of the absorption band associated with LMB (246 nm) and the formation of a hypsochromic shift up to 225 nm. This observation further confirmed that the MB molecules were mineralised during the filtration instead of discoloured^33^. In this case, the injection of electrons into the ZnO nanoparticles on the surface of the filter probably occurred^34^.

**Figure 10:**
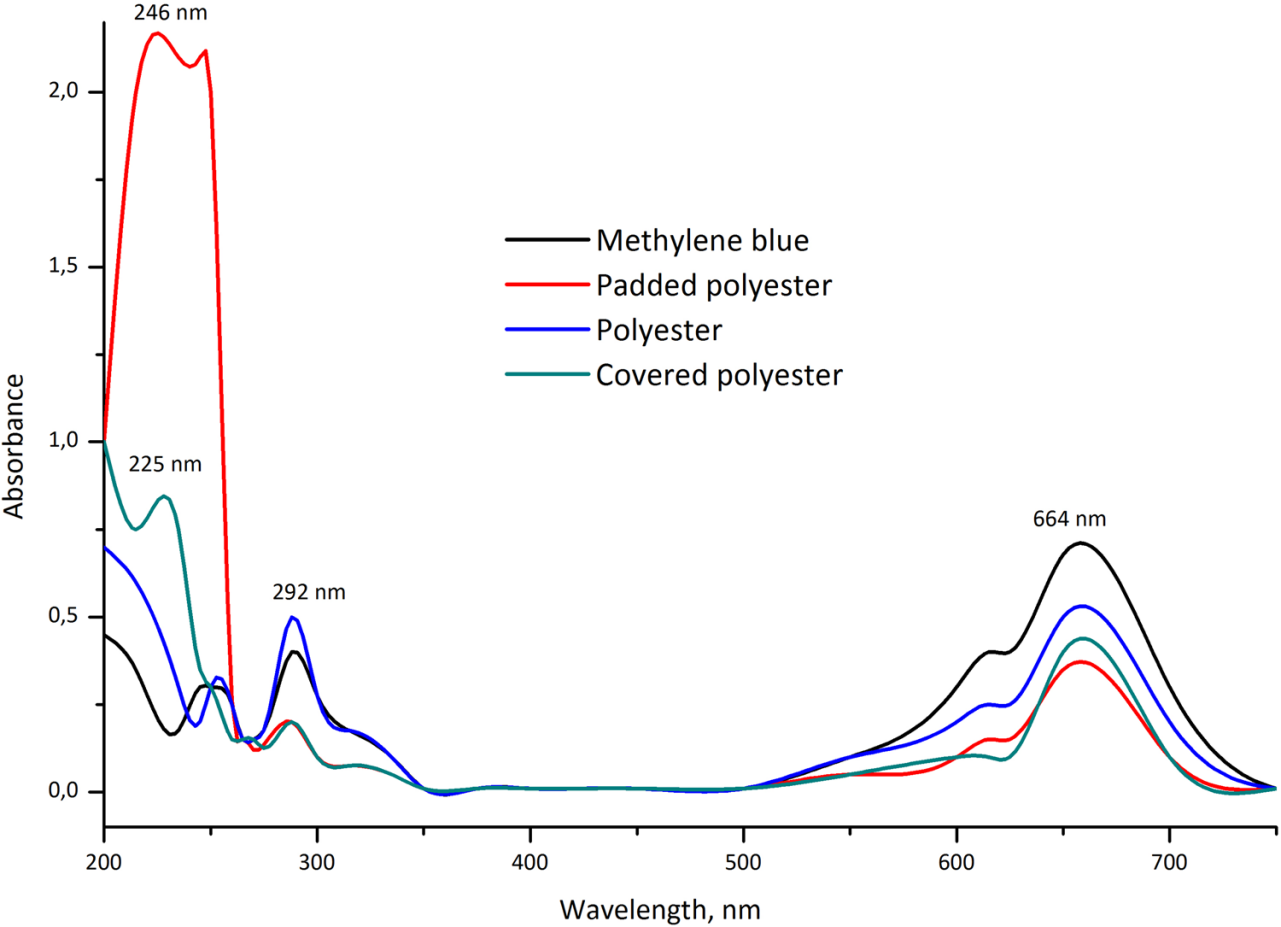
UV/vis spectra recorded for methylene blue solution before and after filtration.

The original blue colour of the dye disappeared and formed a colourless LMB when the padded polyester filter was used. The spectrum shows a steady decrease in the two main absorption peaks (664 and 292 nm) and the appearance of a new band at 246 nm due to the formation of LMB (Fig. 10).

For a pure polyester filter, a decrease in the optical density of the dye was also observed but not significantly. The filters were inactive for MB conversion to LMB. In the absence of the polymer composition on the filter surface, there was no marked decrease in the absorption of the dye.

The problem of detecting MB dye is that the colourless LMB can quickly switch back to the original blue colour of MB through a hydrogenation/oxidation reaction mechanism when the system is exposed to oxygen or air. In the case of developed filters, the styrene-acrylic copolymer can stabilise LMB through hydrogen bonding between the –OH groups of styrene-acrylic molecules and the –N(CH_3_)_2_ groups on LMB and slow down the fast recolouration process (oxidative dehydrogenation process) at ambient conditions^35^. In conclusion, we determined a cost-effective and straightforward process to create two new types of textile nanocomposite filters.

The main findings of this study are listed below:

- New filters were created with suitable thicknesses, lightweight properties, and great flexibility for practical application.
- Microstructure observations revealed that polymer nanocomposite application changed the pore structure in the filter material. Pore diameters decreased with the covered method, which was attributed to the formation of a polymer nanocomposite covering on the surface of polyester textile material. The padded method did not reduce pore size. Moreover, the polymer nanocomposition seeps into all the textile material.
- Due to the hydrophobic property of produced filters, the dye molecules would be absorbed on the surface of the filter, which led to high removal efficiency.
- A colourless reduced form of methylene blue—leuco-methylene blue could be created. The functionalised layer of the developed filters stabilised leuco-methylene blue, keeping it colourless and preventing the conversion back to MB.

## Materials and methods

### Materials

Aqueous dispersion of thermally linking styrene-acrylic copolymer (Tubifast 4010R, CHT, Switzerland: dry residue 45%, pH = 7‒9, viscosity at 20 °C < 500 mPa s) was used as the polymer matrix.

Partially esterified melamine resin (TubifixR, CHT, Switzerland: density 1.15 g/cm^3^, pH = 8.5‒10.5, viscosity at 23 °C 200 mPa s) was used as a crosslinking agent.

Sodium 4-alkyl-2ylbenzenesulfonate (Sulfanol, Germany: dry residue 75%, pH = 8–9) was used as a surfactant (SAS).

ZnO nanoparticles were synthesised by the direct precipitation method^21^ using zinc acetate (Zn(CH_3_COO)_2_·H_2_O) (p.a., 99%, Centralchem, Slovakia) and sodium carbonate (Na_2_CO_3_) (p.a., 99%, Centralchem, Slovakia).

Polyester textile material (China, surface density = 460 ± 30 g/m^2^, bursting load, N length/width = 1100/1200, bursting elongation, % length width = 25/30, breathability = 130 dm^3^/m^2^s, heat resistance 180 °C).

Methylene blue, C_16_H_18_ClN_3_S (MB, 95%, microCHEM). Methyl orange, C_14_H_14_N_3_NaO_3_S (MO, 95%, microCHEM).

### Methods

#### Finishing polyester method

Polymer compositions with ZnO nanoparticles were used to treat polyester fabrics by two methods: one bath pad method and blade coating method. For this purpose, two different compositions were developed (Table 4).

**Table 4:** Prepared polymer nanocomposition.

Composition for one bath pad method	Composition for covering method
Styrene-acrylic copolymer—50%	Styrene-acrylic copolymer 65%
Partially esterified melamine—5%	Partially esterified melamine 25%
ZnO nanoparticles—10%	ZnO nanoparticles 10%
SAS (Sulfanol) 1–35%	

The covering was implemented by the blade coating method. As shown in Fig. S3, the polymer composition was applied to the fabric while being run at tension under a floating knife blade. The distance between the fabric and the knife was 1 mm, which determined the thickness of the coating. The polymer composition had to be highly viscose to prevent soaking through the fabric. The coating was dried at 100 °C for 30 min to fix the covering on the polyester surface. This sample was labelled as covered polyester.

The padding technique, widely regarded as a textile finishing technique, usually refers to a fibre coating of micro or nanomaterials or chemical compositions. As shown in Fig. S4, the fabric was submerged in the polymer composition for 90% pick-up and then dried at 100 °C for 30 min. This sample was labelled as padded polyester.

#### Water absorption test

The sample was dried at 38.5 °C to get a constant weight, noted as W_0_ (g). The samples were then immersed in deionised water for 1 h. took out the fabric and absorbed the surface water droplets with filter papers (the whole process should take no more than 1 min), weighted the fabric and recorded as W_1_ (g). The water absorption capacity of the fabric was calculated by the following equation:

$$W= \frac{({W}_{1}-{W}_{0})}{{W}_{0}}*100.$$

#### Thickness and surface density

The area density PA (g/m^2^) was calculated according to equation:

$${\text{Pa}} = \frac{{\text{m}}}{{\text{A}}}$$

where m is mass (g) measured by an electronic balance, and A is area (m^2^).

Fourier transform infrared (FTIR) spectra of the produced filters were recorded with the help of a spectrometer (Avatar 360, Nicolet) in the range of 500–4000 cm^−1^ (resolution 1.93 cm^−1^, 200 scans, 1 s per scan).

Microstructure investigations were performed by a scanning electron microscope (SEM) (ZEISS Auriga Compact) equipped with EDAX energy-dispersive X-ray spectroscope (EDS) and a field emission scanning electron microscope (MIRA 3 FE-SEM microscope, TESCAN, Czech Republic) equipped with a high-resolution cathode (Schottky field emitter) and with three-lens Wide Field Optics™ design.

### Experimental process

**Figure 11:**
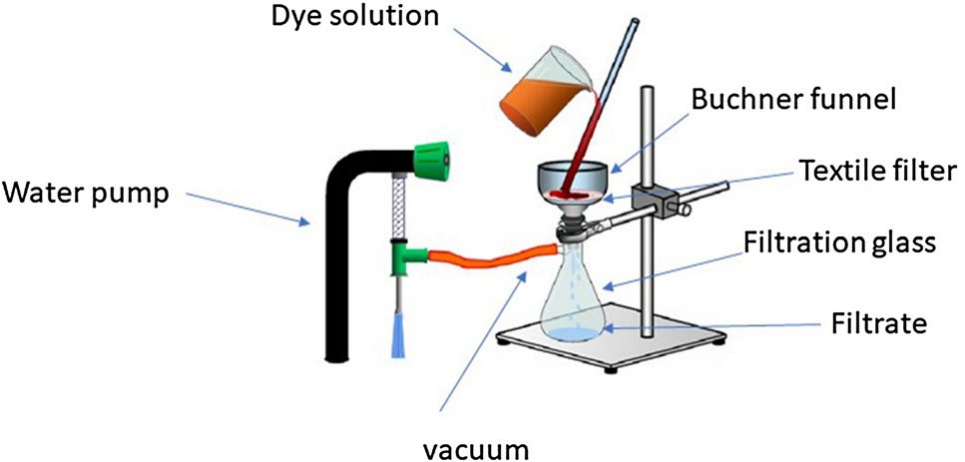
Vacuum filtration method.

A filtration study was carried out with a textile filter when 25.0 mL (100 ppm solutions) of dye at pH ∼ 6.0 in a dynamic mode moved through the filter with an average speed of 0.2 mL/s (120 s). Filtration was conducted by the vacuum filtration method (Fig. 11), using a Buchner porcelain funnel and filtration glass flask. According to this technique, a dye solution was vacuum-filtered through produced filters. The water from the funnel penetrates the filter and flows into a conical beaker by reducing the pressure with a water pump. The experiments were conducted at room temperature. The pressure was constant for all tests. The filtration of the dyes solution was carried out under visible light generated by a 9 W (3000 K) light-emitting diode (LED) lamp located on the top of the laboratory table 40–50 cm above the Buchner funnel (Fig. 11). The filtrate was placed in a spectrophotometer cell immediately after filtration. The concentration of dyes was measured on a Helios Gamma UV–vis spectrophotometer (Thermo electron corporation, UK) for MB in the region 200–700 nm and MO—300–600 nm.

The amount of dye adsorbed onto the fabric surface at equilibrium, *Q*_e_ (mg/g), was calculated by the following expression:

$${Q}_{e}= \frac{({C}_{0}-{C}_{e})V}{Mm}$$

where *C*_0_ and *C*_*e*_ are the initial and equilibrium dye concentrations in mg/L, respectively, *V* is the volume of solution (L), and *m* is the mass of the filter (g).

The filtration efficiency (retention in %) was calculated by the following:

$$R=\frac{({C}_{0}-C)}{{C}_{0}}*100\%$$

where C_0_ is the mass concentration of dyes upstream, and C is the mass concentration of dye downstream.

## Supplementary Information

Supplementary Figures.

## Data Availability

All data generated or analysed during this study are included in this published article (and its Supplementary Information files).

## References

[1] FarhanHanafi M, Sapawe N (2021). A review on the water problem associate with organic pollutants derived from phenol, methyl orange, and remazol brilliant blue dyes. Mater. Today Proc..

[2] Kumar R, Pal P (2012). Response surface-optimized Fenton’s pre-treatment for chemical precipitation of struvite and recycling of water through downstream nanofiltration. Chem. Eng. J..

[3] Power plant in Sweden uses water treatment system incorporating NX Filtration's hollow-fibre NF membranes. *Membr. Technol.* **2021**, 1, 10.1016/S0958-2118(21)00050-1 (2021).

[4] Lv M (2019). Roles of magnetic particles in magnetic seeding coagulation-flocculation process for surface water treatment. Sep. Purif. Technol..

[5] He C (2022). Rational design to manganese and oxygen co-doped polymeric carbon nitride for efficient nonradical activation of peroxymonosulfate and the mechanism insight. Chem. Eng. J..

[6] Zhou C (2021). Strategies for enhancing the perylene diimide photocatalytic degradation activity: method, effect factor, and mechanism. Environ. Sci. Nano.

[7] Zhao H (2019). Robust sandwich micro-structure coating layer for wear-resistant conductive polyester fabrics. Appl. Surf. Sci..

[8] Pasichnyk M, Kucher E, Hyrlya L (2018). Synthesis of magnetite nanoparticles stabilized by polyvinylpyrrolidone and analysis of their absorption bands. Eastern-European Journal of Enterprise Technologies.

[9] Phuong N (2016). Nano sand filter with functionalized nanoparticles embedded in anodic aluminum oxide templates. Sci. Rep..

[10] Jawad AH, Alkarkhi AFM, Mubarak NSA (2015). Photocatalytic decolorization of methylene blue by an immobilized TiO_2_ film under visible light irradiation: Optimization using response surface methodology (RSM). Desalin. Water Treat..

[11] Shinde DR, Tambade PS, Chaskar MG, Gadave KM (2017). Photocatalytic degradation of dyes in water by analytical reagent grades ZnO, TiO_2_ and SnO_2_: A comparative study. Drink. Water Eng. Sci..

[12] Hussein F, Abass TA (2010). Photocatalytic treatment of textile industrial wastewater. Int. J. Chem. Sci..

[13] Semeshko O, Pasichnyk M, Hyrlya L, Vasylenko V, Kucher E (2019). Studying the influence of UV adsorbers on optical characteristics of light-protective polymer films for textile materials. East.-Eur. J. Enterp. Technol..

[14] Han H, Bai R (2010). Highly effective buoyant photocatalyst prepared with a novel layered-TiO_2_ configuration on polypropylene fabric and the degradation performance for methyl orange dye under UV–Vis and Vis lights. Sep. Purif. Technol..

[15] Wang J, Zhao J, Sun L, Wang X (2014). A review on the application of photocatalytic materials on textiles. Text. Res. J..

[16] Hebeish AA, Abdelhady MM, Youssef AM (2013). TiO_2_ nanowire and TiO_2_ nanowire doped Ag-PVP nanocomposite for antimicrobial and self-cleaning cotton textile. Carbohydr. Polym..

[17] Guo M-X, Bian S-W, Shao F, Liu S, Peng Y-H (2016). Hydrothermal synthesis and electrochemical performance of MnO_2_/graphene/polyester composite electrode materials for flexible supercapacitors. Electrochim. Acta.

[18] Meng L (2018). Surface carboxyl-activated polyester (PET) fibers decorated with glucose carbon microspheres and their enhanced selective adsorption for dyes. J. Phys. Chem. Solids.

[19] Xiong G, Pal U, Serrano JG, Ucer KB, Williams RT (2006). Photoluminesence and FTIR study of ZnO nanoparticles: the impurity and defect perspective. Phys. Status Solidi C.

[20] Pasichnyk M, Kucher E (2016). A mathematical modeling of crosslinking between components of a polymer composition. East.-Eur. J. Enterp. Technol..

[21] Pasichnyk M, Václavíková M, Melnyk I (2021). Fabrication of polystyrene-acrylic/ZnO nanocomposite films for effective removal of methylene blue dye from water. J. Polym. Res..

[22] Li X (2019). Waterproof-breathable PTFE nano- and microfiber membrane as high efficiency PM2.5 filter. Polymers.

[23] Zahid M, Mazzon G, Athanassiou A, Bayer IS (2019). Environmentally benign non-wettable textile treatments: A review of recent state-of-the-art. Adv. Coll. Interface. Sci..

[24] Xin Q (2021). Electrospinning in membrane contactor: manufacturing Elec-PVDF/SiO_2_ superhydrophobic surface for efficient flue gas desulphurization applications. Green Chem. Eng..

[25] Jia P, Tan H, Liu K, Gao W (2018). Removal of methylene blue from aqueous solution by bone char. Appl. Sci..

[26] Danish M, Hashim R, Ibrahim M, Sulaiman O (2013). Characterization of physically activated acacia mangium wood-based carbon for the removal of methyl orange dye. BioResources.

[27] Majumdar S, Saikia U, Mahanta D (2015). Polyaniline-coated filter papers: Cost effective hybrid materials for adsorption of dyes. J. Chem. Eng. Data.

[28] Tanzifi M (2018). Adsorption of Amido Black 10B from aqueous solution using polyaniline/SiO_2_ nanocomposite: Experimental investigation and artificial neural network modeling. J. Colloid Interface Sci..

[29] Snehalatha T, Rajanna KC, Saiprakash PK (1997). Methylene blue–ascorbic acid: An undergraduate experiment in kinetics. J. Chem. Educ..

[30] Lee S-K, Mills A (2003). Novel photochemistry of leuco-methylene blue. Chem. Commun..

[31] Galagan Y, Su W-F (2008). Reversible photoreduction of methylene blue in acrylate media containing benzyl dimethyl ketal. J. Photochem. Photobiol. A.

[32] Kadokawa J-I, Izawa H, Ohta T, Wakizono S, Yamamoto K (2011). Photo-induced reduction reaction of methylene blue in an ionic liquid. Int. J. Org. Chem..

[33] Islam MA (2011). Adsorption and UV-visible light induced degradation of methylene blue over ZnO nano-particles. Int. J. Chem. Reactor Eng..

[34] Trandafilović LV, Jovanović DJ, Zhang X, Ptasińska S, Dramićanin MD (2017). Enhanced photocatalytic degradation of methylene blue and methyl orange by ZnO: Eu nanoparticles. Appl. Catal. B.

[35] Liu Y-N (2017). Hydrogenation/oxidation induced efficient reversible color switching between methylene blue and leuco-methylene blue. RSC Adv..

