# Supplementary material for: Development of polyester filters with polymer nanocomposite active layer for effective dye filtration
Source: Sci Rep. 2022 Jan 19;12:973. doi: 10.1038/s41598-022-04829-4 (PMC8770467; doi:10.1038/s41598-022-04829-4)
Supplement: Supplementary file 1 — Supplementary Figures. [file 41598_2022_4829_MOESM1_ESM.docx]

**DEVELOPMENT OF POLYESTER FILTERS WITH POLYMER NANOCOMPOSITE ACTIVE LAYER FOR EFFECTIVE DYE FILTRATION**

**Mariia Pasichnyk^1,2^*, Jana Gaálová^2^, Peter Minarik^3^, Miroslava Václavíková^4^, Inna Melnyk^4^**

^1^ V.О. Sukhomlynskyi National University of Mykolaiv, 24, Nikolska, Mykolaiv 54030, Ukraine

^2^ Institute of Chemical Process Fundamentals of the CAS, v.v.i., 135, Rozvojova, Prague16500, Czech Republic

^3^ Charles University, 5, Ke Karlovu, Praha 12116, Czech Republic

^4^ Institute of Geotechnics Slovak Academy of Sciences, 45, Watsonova, Kosice 04001, Slovak Republic

*Corresponding [pasechnik86@gmail.com](mailto:pasechnik86@gmail.com)


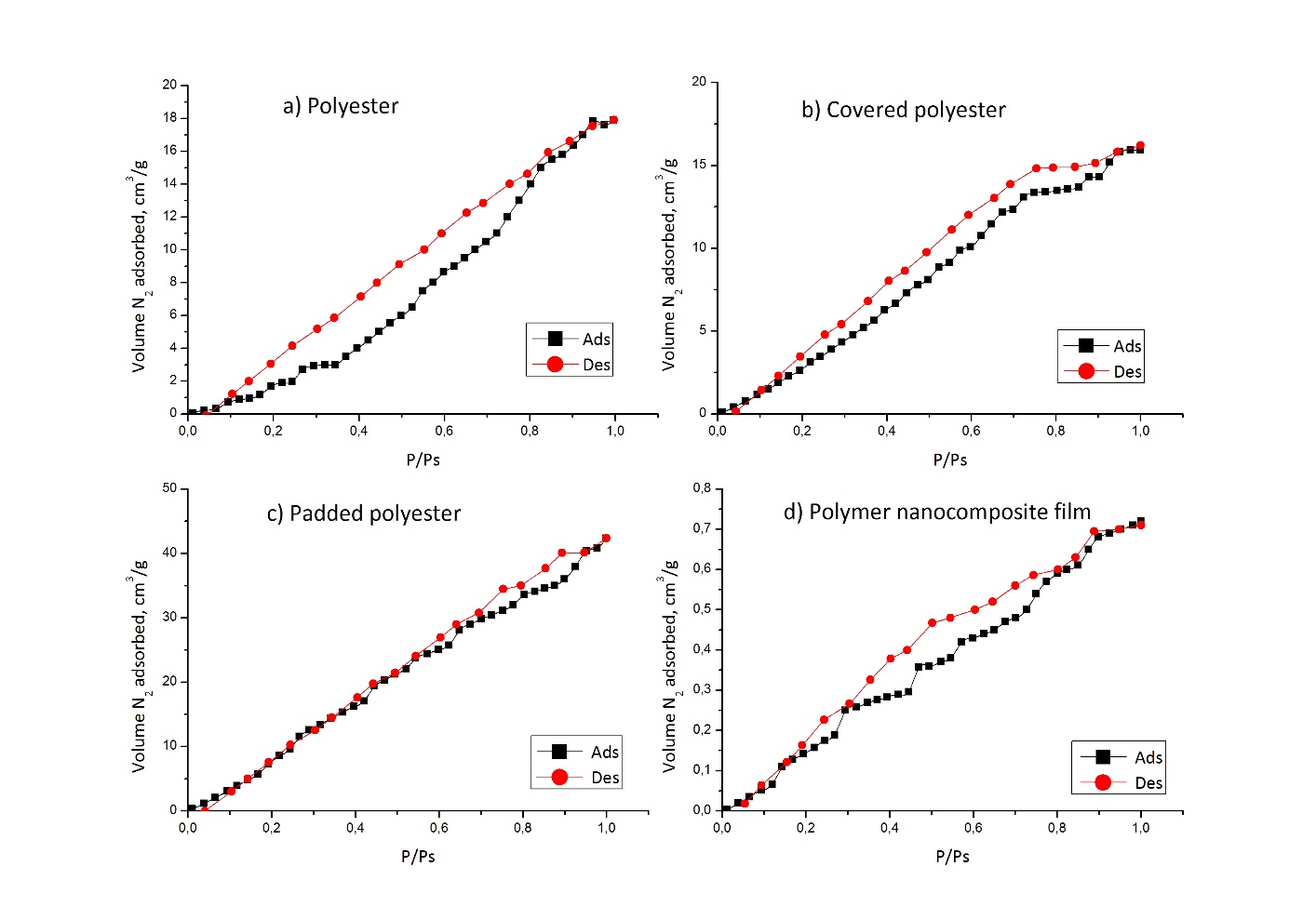


**Figure S1.** Nitrogen adsorption-desorption isotherms

**Figure S2.** Pore size distribution.


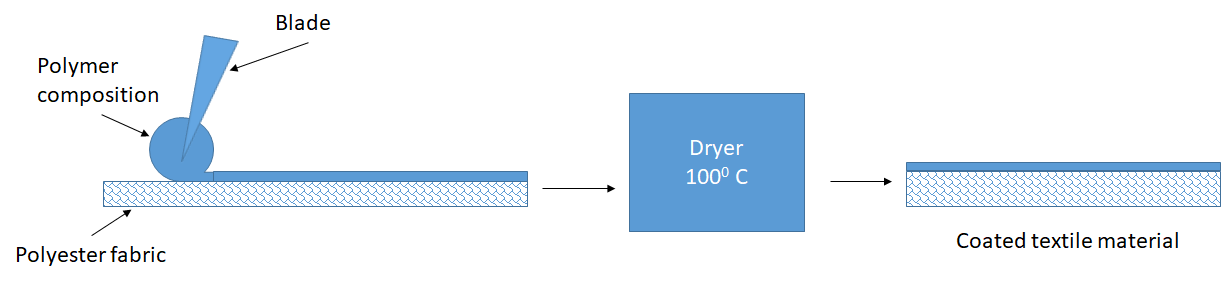


**Figure S3.** The Blade coating method (covering of polyester)


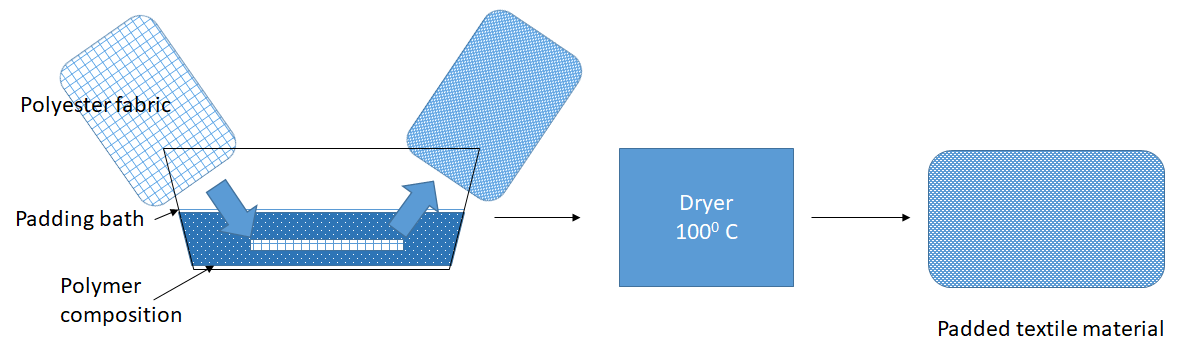


**Figure S4.** One-bath pad method
